# Differential DNA Methylation Patterns Are Related to Phellogen Origin and Quality of *Quercus suber* Cork

**DOI:** 10.1371/journal.pone.0169018

**Published:** 2017-01-03

**Authors:** Vera Inácio, Pedro M. Barros, Augusta Costa, Cristóvão Roussado, Elsa Gonçalves, Rita Costa, José Graça, M. Margarida Oliveira, Leonor Morais-Cecílio

**Affiliations:** 1 Linking Landscape, Environment, Agriculture and Food (LEAF), Institute of Agronomy, University of Lisbon, Lisbon, Portugal; 2 Genomics of Plant Stress Unit, ITQB NOVA—Instituto de Tecnologia Química e Biológica António Xavier, Universidade Nova de Lisboa, Oeiras, Portugal; 3 Instituto Nacional de Investigação Agrária e Veterinária, I.P., Oeiras, Portugal; 4 Center for Environmental and Sustainability Research (CENSE), Environmental Sciences and Engineering Department, Faculty of Science and Technology, NOVA University of Lisbon, Caparica, Portugal; 5 Forest Research Center, Institute of Agronomy, University of Lisbon, Lisbon, Portugal; Institute of Crop Science, CHINA

## Abstract

DNA methylation is thought to influence *Quercus suber* cork quality, which is the main constraint for its economic valorisation. However, a deep knowledge of the cytosine methylation patterns disclosing the epigenetic variability of trees with different cork quality types is totally missing. This study investigates the hypothesis that variations in DNA methylation contribute to differences in cork cellular characteristics directly related to original or traumatic phellogen activity. We used MSAPs (Methylation Sensitive Amplified Polymorphism) to assess DNA methylation patterns of cork and leaf tissues of *Q*. *suber* adult trees growing in three cork oak stands. The relationship between the detected polymorphisms and the diversity of cork quality traits was explored by a marker-trait analysis focusing on the most relevant quality characteristics. Populations differed widely in cork quality, but only slightly in degree of epigenetic differentiation. Four MSAP markers (1.3% of the total) were significantly associated with the most noteworthy quality traits: wood inclusions (nails) and porosity. This evidence supports the potential role of cytosine methylation in the modulation of differential phellogen activity either involved in localized cell death or in pore production, resulting in different cork qualities. Although, the underlying basis of the methylation polymorphism of *loci* affecting cork quality traits remain unclear, the disclosure of markers statistically associated with cork quality strengthens the potential role of DNA methylation in the regulation of these traits, namely at the phellogen level.

## Introduction

Epigenetic variation can contribute to phenotypic plasticity and phenotype persistence in distinct environments, as has been recently suggested in long-lived forest trees (reviewed in [1]). Epigenetics refers to the mitotically and/or meiotically heritable changes in gene expression that do not imply modifications in DNA sequence [2–4]. Chromatin structure is affected by the interplay between DNA methylation, histone modifications and small RNAs, with cytosine methylation being the better-studied chromatin alteration. DNA methylation in plants is accomplished by a family of DNA methyltransferases comprising three specific functional classes [5]: the MET (Methyltransferase) class maintains the methylation in CG context found either within promoter regions [6,7] resulting in silencing [8] or within gene bodies [6,9,10] associated with gene expression [7]; the CMT (Chromomethylase) class maintains the methylation in CHG context occurring mainly in transposons and pseudogenes [10]; and the DRM (Domain-Rearranged-Methyltransferase) class associated with the *de novo* methylation in CHH sequences which are typically methylated at low levels in Arabidopsis [5].

Methylation-sensitive amplified polymorphisms (MSAP) is a practical method that has been widely used to investigate diversity of cytosine methylation and epigenetic structure in species lacking a sequenced genome for reference, as well as to investigate correlations between phenotypic functional traits [11–17]. Also, MSAP markers have been mapped to protein-coding genes in sorghum [18], maize [19,20] and rice [21], and also linked with stable epigenetic quantitative trait *loci* (QTL^epi^) of diverse agronomic traits [22]. Despite the limitations in acknowledging the exact genomic location for methylation and in whole genome coverage, MSAP still provides a significant number of anonymous *loci* randomly distributed throughout the genome in which the methylation state of restriction sites can be determined.

The cork oak (*Quercus suber* L.) is one of the most important forest species in the Mediterranean basin. Cork oak trees are currently the exclusive commercial source of cork, a thick periderm with insulating and protective role, made of dead cells with empty lumens and thin highly suberized walls [23,24]. Cork is formed by the division to the outside of the phellogen, a secondary meristem that is seasonally activated and that persists throughout the life of the tree [23,25]. Cork produced from the original phellogen is called ‘virgin’ cork and is usually harvested from 18 to 25 years-old trees, when stem perimeter reaches the legal size for extraction [26]. After cork removal, the phellogen dies and a new traumatic phellogen is formed by a process of meristematic activation in the underlying non-conducting phloem living cells [27]. Further cork extractions follow at a minimum of 9-years intervals, during which a cork thickness of 2–6 cm is reached. This capacity for self-regeneration of the phellogen determines cork oak uniqueness and makes cork the cornerstone of the economic sustainability of cork oak woodlands worldwide. *Amadia* cork, produced by a traumatic phellogen (3^rd^ harvest onward) has singular characteristics derived from cellular structure and chemical composition. However, as a natural product, its quality criteria is determined by the cork tissue homogeneity and thickness, due to their industry applications, for some of them without viable substitutes [28].

Cork tissue homogeneity is affected by the incidence of discontinuities in the cork layer, such as lenticels (forming the lenticular channels) produced by the activity of the lenticular phellogen, and lignified phloem cells, known as ‘nail’ structures [29] and caused by an interrupted, abnormal phellogen activity which is highly detrimental to cork quality. In addition, cork thickness is mostly determined by the level of phellogen activity and corresponding cumulative annual cork growth (cork-ring width). A minimum thickness is needed in the raw cork planks in order to allow punching natural cork stoppers, the most valuable cork product. Despite these critical quality parameters of raw cork, the disclosure of factors regulating cork differentiation and quality at tree-level is in its infancy [30,31]. The high genetic variability found within cork oak populations [32,33], mixed with empirical assumptions of an effect of environmental conditions, hindered any causal association with the very high cork quality variability found within and between cork oak woodlands [29]. To better understand what affects cork quality, it is necessary to unravel the factors underlying such different phellogen activities giving rise to such variable phenotypes. Recent studies of the epigenetics of oak populations revealed patterns of epigenetic differentiation and single-methylation variants associated with climate variables [34,35], indicating that oak genomes exhibit phenotypic plasticity mediated by DNA methylation. Although the genus *Quercus* has already two sequenced genomes [36,37] no genomic data was available for cork oak. Some studies have been targeting cytosine methylation in this species [38–40] evidencing its association with cork quality, namely on cork tissue homogeneity [39].

Considering the role of DNA methylation in regulating phenotypic plasticity we hypothesised that DNA methylation variability could be related to original or traumatic phellogen activity, also contributing to cork cellular characteristics linked to quality. A deeper understanding of cytosine methylation variability at tree-level and its impact on relevant traits could lead to a better comprehension of its role in the modulation of cork tissue homogeneity. To test our hypothesis, we assessed the DNA methylation landscape of virgin and *amadia* cork tissues and leaves, focusing on CG and CCG context using MSAP analysis, in trees from three regions in Portugal with distinct edaphoclimatic conditions. Furthermore, to test for predicted relationships between the variation in DNA methylation and phenotypic diversity, the epigenetic polymorphisms detected were associated with the most relevant cork quality traits.

## Materials and Methods

### Plant material and DNA isolation

No specific permission was needed for the development of this study. *Quercus suber* is protected in Portugal against logging. The field studies did not involve endangered or other protected species.

Cork oak adult trees were randomly chosen from three cork oak stands (*montados*) in different locations in Portugal: Barradas da Serra (BS), Herdade dos Leitões (HL) and Companhia das Lezírias (CL) (Table 1). Each stand comprised trees of controlled origin as well as trees resulting from natural regeneration. A detailed characterization and comparison of the three areas, in terms of climate and soil conditions, is described elsewhere [41]. Cork planks were harvested at breast height (at 1.30 m height from soil) during the more intense period of phellogen activity (the period of cork commercial harvesting), and phellogen with contiguous differentiating tissue (hereafter referred as cork tissues) were collected by scraping off the inner side of cork planks. Leaves were also collected from all trees. Cork planks were kept to evaluate cork quality and thickness. To avoid any developmental, and/or environmentally-related variation in DNA methylation, fully developed leaf blades and cork tissues were collected from each location on the same day. All living tissues were immediately stored in liquid nitrogen until further use. Total genomic DNA from leaves, and cork tissues was isolated according to Doyle and Doyle [42] with minor modifications: the isolation buffer contained 3% 2-mercaptoethanol and 2% PVP-40; proteinase K was added to a final concentration of 100 μg/mL before samples incubation at 65°C.

**Table 1:** Material collected in three cork oak populations in distinct edaphoclimatic conditions in Portugal.

Populations	Geographical Coordinates	Trees^b^		
		*Amadia* cork	Virgin cork	Total
Grândola, Barradas da Serra (BS)	38 11’ N, 8 37’ W 270 m a.s.l.^a^	8	1	9
Montargil, Herdade dos Leitões (HL)	39 8’ N, 8 11’W 170 m a.s.l. ^a^	11	0	11
Benavente, Companhia das Lezírias (CL)	38 49’ N, 8 49’ W 20 m a.s.l. ^a^	10	11	21

^a^ Above sea level

^b^ Number of individuals sampled per population

### MSAP procedure

MSAP is a modification of the AFLP method, which takes advantage of the differential behaviour of two isoschizomers, *Hpa*II and *Msp*I, in the presence of cytosine methylation in the CCGG context. These isoschizomers are coupled with *Eco*RI, which is thought to be negligibly influenced by DNA cytosine methylation. *Msp*I can cleave non-methylated CCGG sequences and hemi- or fully-methylated C^m^CGG sequences but not hemi- and fully-methylated ^m^CCGG and ^m^C^m^CGG sequences. *Hpa*II digests only non-methylated CCGG sequences and hemi-methylated ^m^CCGG sequences from all possible methylated CCGG variants [43]. Therefore, distinct profiles obtained with *Eco*RI/*Hpa*II and *Eco*RI/*Msp*I reflect differences in the methylation status of these restriction sites.

For MSAP assays, genomic DNA (100 ng) isolated from cork tissues and leaves of the same genotype was first digested and ligated using 20 U of *Eco*RI and 5 U of *Msp*I or *Hpa*II (New England Biolabs, USA), 5 pmol *Eco*_adaptor, 50 pmol HM_adaptor (5 μM of *Eco*RI and 50 μM of *Msp*I/*Hpa*II adaptor pairs were previously prepared by mixing oligonucleotides and incubating at 98°C for 5 min followed by slow cooling in aluminium foil–S1 Table), 1 U of T4 DNA ligase (Invitrogen, USA) in 40 μL total volume supplemented with 50 mM NaCl and 100 ug/ml BSA for 6 h at 37°C. The enzymes were then inactivated by heating to 65°C for 15 min. A volume of 2 μL of the restriction/ligation products was used in 20 μL pre-selective amplification reactions containing 1X PCR buffer (Nzytech, Portugal), 30 ng of Pre_*Eco* (+A) primer and 30 ng of Pre_HM primer (S1 Table), 0.4 mM dNTPs, 1.5 mM MgCl_2_ and 2 U Taq polymerase (Nzytech, Portugal). PCR conditions were 2 min at 72°C followed by 25 cycles of 94°C for 1 min, 56°C for 1 min and 72°C for 2 min with a final extension step of 10 min at 72°C. Initially, 25 selective primer combinations were tested for fitness in identifying inter-specific variation and in generating reproducible MSAP profiles using two independent DNA extractions of each tissue from four representative genotypes (data not shown). From these, four primer combinations (S2 Table) were chosen for the comparative selective amplification. Selective PCRs were then performed using 5 μL of 1:10 dilution of pre-selective PCR products and the same reagents as the pre-selective amplification, but using 0.2 mM dNTPs and FAM labelled selective primers. The conditions of the touch-down selective PCR were as follows: 94°C for 30 s, 65°C for 30 s, 12 cycles of 72°C for 1 min (decreasing by 0.7°C each cycle), followed by 24 cycles of 94°C for 30 s, 56°C for 1 min, 72°C for 2 min, ending with 72°C for 5 min. Thereafter, 1 μL of selective amplification products was mixed with 0.3 μL of ROX dye and 12.2 μL of HiDi formamide (Applied Biosystems, USA), denatured by heating at 95°C for 5 min and snap cooled on ice for 5 min. Fragment separation and detection was made using an ABI PRISM 310 Genetic Analyser (Applied Biosystems, USA) and fragments were scored manually analysing the electropherograms with GENEMAPPER 3.7 software. For each primer combination, genotyping error rates were estimated through the comparison of two technical replicates profiles. MSAP genotyping error rates were estimated for each primer combination by running repeated *Eco*RI/*Hpa*II and *Eco*RI/*Msp*I analyses for all plants and both tissues and computed as described elsewhere [44]. Mean genotyping error rate (±SD) for the four MSAP primer combinations used was 0.004% ± 0.002. A combined digestion with *Eco*RI/*Hpa*II + *Msp*I was performed in parallel to improve the interpretation of the (absence, presence) pattern obtained for some *epiloci* in (*Eco*RI/*Msp*I, *Eco*RI/*Hpa*II) [43]. This approach helps to distinguish between the two situations that may generate that pattern: the cutting of hemimethylated sites mCCGG sites by *Hpa*II and not by *Msp*I; or the presence of internal CmCGG site(s) between the *Eco*RI site and the cleaved distal unmethylated CCGG [43].

### MSAP data analysis

Reproducible peaks with lengths between 100 and 500 bp (to minimize the incidence of fragment size homoplasy [45]) were scored as presence (1) or absence (0) to form a raw data matrix. All *epiloci* showing a monomorphic pattern or any fragment present/absent in all but one individual were excluded from the data set, to prevent biased parameter estimates [46]. ‘Mixed Scoring 2’ scheme and the ‘Extract_MSAP_epigenotypes’ R function described by Schulz *et al*. [44] was used to convert the raw matrix (S3 Table) into three classes of markers corresponding to unmethylated (u-*loci*), ^HMe^CG + ^Me^CG methylation (internal methylation plus hemimethylation, m-*loci*) and ^HMe^CCG methylation (external hemimethylation, h-*loci*). U-*loci* markers displaying the same profile either for *Eco*RI/*Msp*I or *Eco*RI/*Hpa*II in leaves and phellogen from the same genotype were considered as genetic variation and were removed from the epigenetic variability analysis (40 *loci*).

In order to measure the epigenetic diversity within leaves and cork tissues ‘MSAP_calc.R’ script [44] was used to calculate the number and percentage of polymorphic *epiloci* and Shannon’s information index for a dominant locus:

$$H^{\prime }=-(p_{i}\;{log}_{2}\;p_{i}+{(1-p_{i})log}_{2}\left(1-p_{i})\right)$$ (1)

where *p*_*i*_ is the frequency of the epigenetic marker presence per tissue. To test the null hypothesis that the distribution of Shannon´s information index is identical for both tissues, a Mann-Whitney-Wilcoxon test was performed in R environment. To infer patterns of individual and population epigenetic differentiation, a similarity matrix was assembled using the Soerensen and Dice distance and used to conduct the principal coordinate analysis (PCoA).

The epigenetic differentiation was estimated through Φ_st_ comparisons (an analogue of the fixation index Fst, measuring population differentiation) between leaves and cork tissues and among populations for both tissues using an analysis of molecular variance (AMOVA, [47]) and the same Soerensen and Dice similarity matrix. All these analyses were performed using R environment.

### Cork quality traits assessment

*Amadia* cork planks were processed according to a standard procedure [48] to assess cork quality traits but virgin corks were not scorable by the methods used. *Amadia* cork thickness was measured according to Ramos *et al*. [39]. Image acquisition of the two radial, two transversal and one tangential sections was made through scanning at a minimum resolution of 300 dpi. Images were uploaded and then analysed using ImageProPlus® (Media Cybernetics, USA) image-processing software.

In each cork sample, after a calibration based on an orthogonal position correction with an accuracy of 0.01 mm, the detection of cork tissue discontinuities was made based on threshold manipulation within a defined area of interest (AOI). Overall cork porosity was assessed at the tangential section, transverse and radial sections. However, only at transversal and radial sections, the overall porosity was discriminated by porosity (lenticels as lenticular channels, S1 Fig) and ‘nail’ (lignified phloem cells, S2 Fig). Cork tissue discontinuities datasets were obtained separately for each section, and based on the range variation of pore-variables. A set of four variables at cork sample/section level were selected for quality assessment: average pore area, average pore length, average pore roundness and average ‘nail’ area. Pore and ‘nail’ data were filtered out by area, and only cork tissue discontinuities with an area equal or superior to 0.8 mm^2^ were kept for analysis. Small porosity is functionally irrelevant and increases variance and variability of the sample [49]. Pore roundness is defined by the following formula:

$$\frac{{perimeter}^{2}}{4\times \pi \times area},$$ (2)

where circular discontinuities will have roundness ≈ 1. When the pore is less circular the roundness value will be higher. The pore length is the length along the Y-axis (corresponding to the tree radial direction in the transverse and radial sections). At cork section level, the porosity coefficient and the nail coefficient were expressed as the percentage of the total pore and nail area of the AOI.

Assessment of cork ring widths was made in *amadia* cork samples according to a standard procedure [48], measuring the complete cork growth years in the transverse sections. The mean annual cork growth (mm yr^-1^) was then calculated per cork sample.

The population effect on the variability of cork quality traits was studied after testing for deviations from a normal distribution with a Shapiro-Wilk test. For traits that fitted a normal distribution, the effects of the population were evaluated using one way ANOVA tests, followed by Tukey´s multiple comparison test. For non-normally distributed data, Kruskal-Wallis non-parametric and Dunn´s Multiple Correction post-hoc test were used. To find if cork quality traits could be correlated, Pearson’s correlations between traits were computed. All these analyses were performed in R environment.

### Association analysis

To assess whether observed differences in the considered cork quality traits within populations were related to DNA methylation polymorphisms, generalized linear models were fitted to higher cork quality presence data. For such approach, all cork quality traits measured were allocated into 2 classes—higher cork quality and lower cork quality—by applying a defined threshold according to trait distribution and taking into account their shape in each section (Table 2). For the response variable cork quality a logit link model was fitted assuming marker as a fixed-effects factor with two levels (presence/1 or absence/0). To test the null hypothesis of no effects of the marker, a likelihood ratio test was performed. The resulting *p*-values were used to identify significant associations (significance level = 0.05). False discovery rate adjusted *p*-value (*q*-value) were computed using the *qvalue* package [50]. We found the largest *q*-value leading to an expectation of less or equal to one false significant model [i.e. *q*-value x (number of models accepted as significant) ≤1]. For the interpretation of results, the estimated probability of success (higher cork quality, ${\hat{p}}_{1}$) for the MSAP marker presence was computed from the inverse link function. Additionally, for the presence of MSAP marker, the estimated odds that higher cork quality is obtained, was computed as

p^11−p^1. (3)

All these analyses were performed using R environment.

**Table 2:** Thresholds of cork quality traits used for the association study with MSAP markers.

Cork Quality Trait	Threshold ^a^	Reference	Higher cork quality	Lower cork quality
Thickness	27 mm	[49]	Equal or above threshold	Below threshold
Annual growth	3 mm/year	[49]		
% ‘nail’ _R	1%	Outliers	Below threshold	Equal or above threshold
% ‘nail’ _Tr	1%	Outliers		
‘nail’ Area_R	3.98 mm^2^	Median		
‘nail’ Area_Tr	3.99 mm^2^	Third Quartile		
% Porosity_Ta	6%	[49]		
% Porosity_R	6%	[49]		
% Porosity_Tr	6.8%	[49]		
Pores Area_Ta	2.57	Median		
Pores Area_R	5 mm^2^	[51]		
Pores Area_Tr	4.9 mm^2^	[51]		
Pores Roundness_Ta	1.98	Median		
Pores Roundness_R	4.03	Median		
Pores Roundness_Tr	4.56	Median		
Pores Length_Ta	2.5	Median		
Pores Length_R	5 mm	[51]		
Pores Length_Tr	5 mm	[51]		

^a^ Cork quality traits measured in all sections were allocated into 2 classes—higher cork quality and lower cork quality—by applying a defined threshold according to trait data distribution or previously reported values, and taking into account their shape in each section.

R—radial section; Tr—transverse section; Ta–tangential section.

## Results

### Epigenetic diversity and differentiation is higher in cork tissues

**Fig 1:**
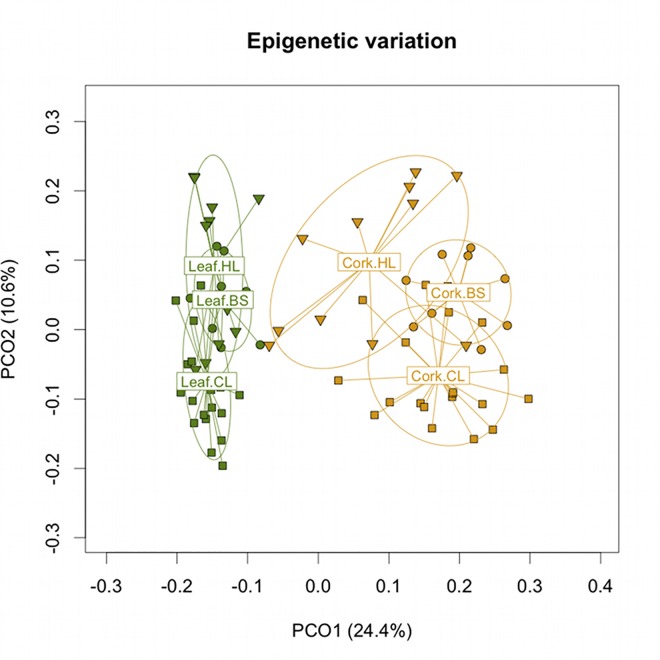
Principal Coordinate Analysis representing epigenetic differentiation between populations and tissues. Graphical representation is based on the first two coordinates (PCO1 and PCO2) with the percentage of the variability shown between brackets. Inverted triangles represent trees from Herdade dos Leitões (HL), circles represent Barradas da Serra (BS) and squares represent Companhia das Lezírias (CL). The labels represent the centroid for the points cloud in each population and the ellipses represent the dispersion of those points around the centroid.

The methylation states of the scored *loci* were obtained after data transformation, yielding a total of 339 polymorphic *epiloci* for cork tissues (out of 340) and 303 polymorphic *epiloci* for leaves (out of 308) (Table 3). The Shannon’s information indexes for each marker were differentially distributed between the two tissues (W = 83102, *p*-value = 0.007749, Wilcoxon rank sum Test). Indeed, a higher number and frequency of u-*loci* was found for cork tissues among all individuals in contrast with a similar number but higher frequency of m-*loci* in leaves. The number of *h*-loci was higher in cork tissues but the frequency was higher in leaves. Comparing both tissues using total polymorphic *loci*, we found significant epigenetic differentiation (Fig 1, Φ_st_ = 0.3321, *p*-value < 0.0001), probably derived from their different nature i.e. meristematic vs. differentiated tissues. Comparing populations, epigenetic differentiation was found for cork tissues Φ_st_ = 0.168 (*p*-value < 0.0001) and for leaves Φ_st_ = 0.1608 (*p*-value < 0.0001).

**Table 3:** Epigenetic diversity of cork and leaf tissues within the three cork oak populations.

Tissue	u-*loci*	m-*loci*	h-*loci*	Total PL
	No./%PL	No./%PL	No./%PL	
**Cork tissues**	161/34.1	106/27.3	72/12.5	339
**Leaves**	133/25.1	108/37.5	62/10.2	303

u-*loci*–unmethylated *loci*; m-*loci–*methylated *loci*; h-*loci–*hemi-methylated *loci*; No.–number of polymorphic *loci*; %PL–Percentage of polymorphic *loci*; Total PL*–*Number of total polymorphic *loci*.

### Epigenetic differentiation in cork tissues relates to phellogen origin

**Fig 2:**
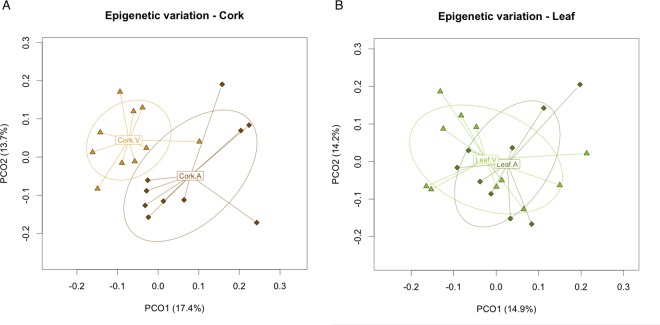
**Principal Coordinate Analysis representing epigenetic differentiation in cork (A) and leaf (B) tissues, between trees producing virgin cork (V) and *amadia* cork (A) from CL population.** Graphical representation is based on the first two coordinates (PCO1 and PCO2) with the percentage of the variability explained shown between brackets. Triangles represent ‘virgin’ cork producing trees and diamonds *amadia* cork producing trees. The labels represent the centroid for the points cloud in each population and the ellipses represent the dispersion of those points around the centroid.

To investigate if phellogen origin (original or traumatic), and/or phellogen age could influence the trend of epigenetic differentiation, we analysed separately the *amadia* cork-producing trees (S3 Fig). Comparing with the whole sample results, a reduced epigenetic divergence between populations was found for cork tissues (Φ_st_ = 0.1432, *p*-value < 0.0001), indicating that virgin-producing trees could be contributing for higher population epigenetic diversity in these tissues. Considering these results, we further investigated whether virgin and *amadia* cork could be differentiated at the epigenetic level, using only leaf and cork tissues from CL population, where 11 trees produced virgin cork and 10 produced *amadia* cork. PCoA showed some epigenetic differentiation for cork samples, according to tissue type (Fig 2A) whereas no clear organization was found for leaves (Fig 2B). Significantly higher epigenetic differentiation was found for virgin cork tissues (Φ_st_ = 0.1196, p- value < 0.0001) when compared to leaves from the corresponding trees (Φ_st_ = 0.0271, *p*-value = 0.1598). These results suggest that the tissues generated from the original phellogen (originating virgin cork), although having similar frequencies of the three types of *loci* (u, m and h), may have different epigenetic marks as compared to the young traumatic phellogen formed after cork removal.

### Cork quality traits are highly variable within populations

**Fig 3:**
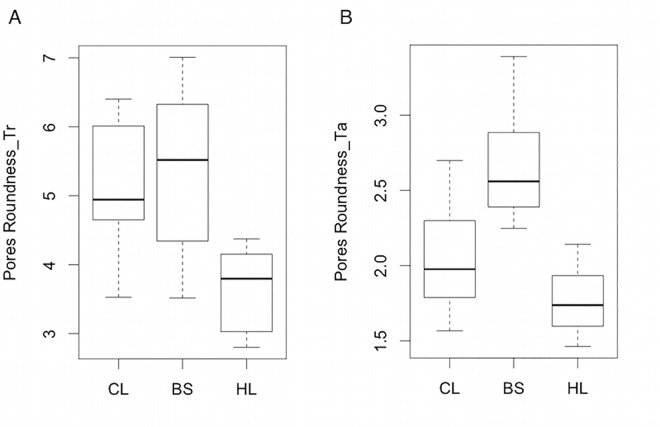
Boxplots representing the distribution of pores roundness. (A) in transverse section (Tr) and (B) tangential section (Ta) in samples from *amadia* cork planks collected from the three populations: Companhia das Lezírias (CL), Barradas da Serra (BS) and Herdade dos Leitões (HL).

Comparing the cork quality traits assessed in the three populations, there was no statistically significant difference in the average values determined for almost all cork quality traits evaluated in each of the three different sections. In fact, most of the variability observed occurred within the populations rather than among them, as denoted by the larger coefficients of variation (S4 Table). One of the exceptions regards the pores roundness assessed in transverse sections (where pores should appear as rectangular channels). In fact, cork from HL population significantly differed from the other two populations (*p*-value = 0.0004, Fig 3A) due to its lower roundness values, which could be associated with shorter lenticular channels radially crossing the cork tissue. Interestingly, in radial sections this difference did not reach statistical significance. Moreover, in tangential sections (where pores should appear with an approximately circular form), corks from BS population displayed pores with a more irregular shape (higher values of roundness), as compared to corks from CL and HL populations (*p*-value < 0.0001, Fig 3B). Another exception concerns the pores’ length in corks from BS population, which was slightly higher than in HL population (*p*-value = 0.0209). This might be due to more elongated (elliptical) shape of lenticular channels in their tangential section, generally associated with lower cork porosity.

### The occurrence of ‘nail’ is negatively correlated with cork growth

Considering the comprehensive amount of data collected for several cork traits determining quality, we have also assessed correlations between them (S4 Fig, S5 Table). In addition to the expected correlation between aspects of the same trait in transverse and radial sections, we also found a predictable strong positive correlation between cork thickness and annual growth (r = 0.875, *p*-value = 5.78e-07). Significant positive correlation was also found between cork thickness and pore area and pore length, in transverse sections, revealing that lenticular phellogen producing pores follow the same annual regularity as phellogen. Interestingly, we found a significant negative correlation between ‘nail’ parameters and cork thickness/ annual growth, suggesting that the localized death of phellogen (creating ‘nails’) has a negative impact on cork growth. This was particularly evident in transverse sections, in which the average area of ‘nail’ and cork thickness or annual growth showed a moderate correlation of -0.501 (*p*-value = 0.0065) or -0.434 (*p*-value = 0.0211), respectively. For ‘nail’ percentage, in the same section, the moderate correlation with cork thickness or annual growth was -0.4624957 (*p*-value = 0.01321) or -0.462 (*p*-value = 0.013), respectively.

### MSAP markers are associated with cork quality traits

The association study among the 315 MSAP polymorphic markers found for *amadia* cork tissues and the considered cork quality traits revealed 7 significant associations (Table 4). These significant marker-trait associations included 4 anonymous *loci* (1.3% of the total markers tested per trait), one of which in two different states of methylation and 3 of the studied traits that mostly contributes to cork quality: annual growth porosity and ‘nail’ presence. One MSAP marker (hemi-methylated C4_487) was associated with the ‘nail’ percentage in transverse sections, that when present there is an estimated probability that 95% of the individuals have a low percentage of ‘nail’ (high cork quality). Another interesting association was found between the C1_175 MSAP marker, that can be present either in methylated or unmethylated state, and the porosity coefficient and pores’ area. When this sequence is methylated, the estimated probability of corks showing lower pore area (high cork quality) is 94%.

**Table 4:** Statistically significant logistic regressions relating cork quality traits and MSAP marker presence.

MSAP marker	Restriction site methylation state	Cork quality trait	Significance of MSAP marker effect ^a^	*q*-value ^b^	${\hat{p}}_{1}$^c^	Odds ^d^
			*p*-value			
C4_487	Hemi-methylated	% ‘Nails’_Tr	0.00094	0.0681	0.95	20.0
C1_175	Unmethylated	Pore area_Tr	0.00020	0.0140	0.31	0.44
		% Porosity_R	0.00054	0.0237	0.12	0.14
	Methylated	Pore area_Tr	0.00020	0.0140	0.94	15.0
		% Porosity_R	0.00054	0.0237	0.50	1.00
C2_101	Unmethylated	Pore area_Tr	0.00002	0.0040	0.27	0.36
C1_296	Unmethylated	Pore area_Tr	0.00056	0.0293	0.18	0.22

Generalized linear models were fitted to higher cork quality presence data browsed for the N = 29 amadia cork samples (see Table 3 for details), using a logit link model and assuming fixed effects for MSAP marker.

^a^ Likelihood ratio tests to verify the null hypothesis of no effects of the marker were performed.

^b^ Largest q-value leading to an expectation of less or equal than one falsely significant model [i.e. q-value x (number of models accepted as significant) ≤1].

^c^ Estimated probability of success (higher cork quality), when MSAP marker is present.

^d^ The estimated odds to obtain higher cork quality (success) when the marker is present is ${\hat{p}}_{1}/1-{\hat{p}}_{1}$.

R—radial section; Tr—transverse section

## Discussion

In this study differences in DNA methylation patterns in virgin and *amadia* corks, and in leaf tissues of *Q*. *suber* adult trees were analysed. The comparison of DNA methylation profiles of cork and leaves, two different tissues from the same genotype, allowed differentiating genetic from epigenetic polymorphisms. The detected epigenetic polymorphisms were further associated with the most relevant cork quality traits: growth, porosity and presence of ‘nails’. MSAP was the method chosen for this study although the impossibility in identifying the sequence, location in the genome and the genes affected by the methylation. Regardless of these limitations we were able to assess the diversity of DNA methylation patterns in a significant number of CG and CCG *loci*, as well as to investigate associations with phenotypic traits, in a species without sequenced genome. Moreover, the MSAP scoring used in this study and previously suggested by Schulz and colleagues [44] allowed for scoring either methylated, unmethylated or external hemimethylated fragments, providing an extended view of genome methylation, that single scoring methods do not allow.

### Epigenetic differentiation in cork stands

In order to differentiate methylated from mutated *loci*, cork tissues and leaves from the same individuals were used. However, this information could be achieved only for u-*loci*, when both *Hpa*II and *Msp*I were used for DNA digestion and equal MSAP profiles were found for both tissues in the same genotype. Thus, any difference in MSAP profiles between distinct genotypes should reflect mutated *loci* which were kept out of the analysis due to its reduced number, comparing with the number of *epiloci* found. To our knowledge, this is the first work making use of this approach in trees, to reduce some bias due to genetic variation.

Cork and leaf tissues showed a higher frequency of ^m^CG methylation (m-*loci*) compared to ^m^CCG (h-*loci*) in agreement with the overall methylation frequencies found in plant genomes [10,52]. Still, the frequency of all methylated *loci* (m-*loci* plus h-*loci*) was higher in leaves than in cork tissues and the opposite was found for unmethylated *loci*. However, it must be highlighted that MSAP does not detect all methylation contexts or fully methylated external sites associated with plant heterochromatin [44,52]. Nevertheless, these results may arise from the different nature of both tissues, as cells were fully differentiated in leaves while meristematic activity is found in the phellogen of cork tissues. Indeed, since fully differentiated cells in cork are devoid of cellular content [24], cork samples include mostly meristematic cells (in the phellogen most intense activity period) and contiguous young cells at early differentiation stage [53,54]. Also, the distinct cell differentiation stages present in these two tissues might contribute to the significantly higher cytosine methylation diversity found in cork tissues, as also observed in juvenile or undifferentiated *Pinus* tissues [55,56].

The three cork oak populations analysed showed a relatively high level of epigenetic differentiation (Φ_st_ = 0.16) for either leaf or cork samples. Previous work in *Q*. *lobata*, using reduced-representation bisulphite sequencing reported higher levels of population differentiation at CG contexts (*F*_*ST*_ = 0.28) than at CHG contexts (*F*_*ST*_ = 0.08) [34]. Differences in epigenetic differentiation between cork and valley oaks can be explained by the distinct methods used: for cork oak, CG and CCG contexts were evaluated together while for valley oaks these two contexts were used separately. Still, our result may reflect some degree of local adaptation related to polymorphic *epiloci*, as also suggested for valley oaks [34], probably due to stable epialleles appearing during these trees’ long life on the same environment. Nevertheless, most of the variability was found within populations which is consistent with the patterns observed also for the genetic variability of this oak populations [32,33] and for cork quality [29,57]. Heritable DNA methylation variants can arise spontaneously in the absence of genetic control [58,59] and potentially affect adaptation. However, it remains undetermined if the epigenetic variation found here is autonomous or an effect of underlying genetic variation, given the cork oak high genetic variability.

### DNA methylation patterns differ according to the phellogen origin and age

Our results indicate that virgin and *amadia* corks show distinct DNA methylation profiles, leading to some degree of epigenetic differentiation, while no variation was found in leaves of the same trees. After formation, phellogen remains seasonally active throughout the life of the tree and produces virgin cork for several years. When cork is removed the original phellogen dies and a traumatic phellogen is formed in response to the wounding stimulus, by a process of meristematic activation of the non-conducting phloem living cells [28,60]. Therefore, virgin and *amadia* corks have distinct origins and develop from phellogens with different ages, since the traumatic phellogen has nine years old whereas the original phellogen has at least 25 years of age. Younger phellogens have been reported as having higher growth activity [48], and methylation profiles can be determined by underlying mechanistic differences linked to ageing, as seen in other plants [55,56,61]. Another hypothesis is related to the different origin and growth activity of original and traumatic phellogen. Plants have mechanisms to regenerate tissues via dedifferentiation or transdifferentiation, as seen in the xylem activation of meristematic activity during vascular repair (reviewed in [62]). Genes involved in chromatin modifications and remodelling, together with cell cycle genes were found to be up-regulated during the early regeneration process after bark girdling in *Populus* [63]. These results reveal that the capacity of xylem cells to change their fate is modulated by epigenetic regulation when cell cycle is activated [63]. Considering this, we may also hypothesize that after traumatic chromatin remodelling, some “memory” may have been imprinted in the newly formed phellogen, thus accounting for differences in methylation patterns between *amadia* and virgin cork tissues.

### The presence of ‘nails’ is associated with DNA methylation

The extent of cork defects measured in this study, as well as the variability found among populations are within the values reported in previous publications [29,57]. Although the origin of these cork traits can be explained in the context of development, the genes controlling their frequency or shape are unknown. In this work we found a negative association between cork growth parameters (thickness and average annual growth) and the percentage of nail inclusions.

The width of the annual growth ring has been attributed to the number of cells produced by each phellogen mother-cell [64]. In turn, ‘nails’ are formed when small portions of the phellogen die and a new meristematic capacity arises in living cells inside the underlying phloem, isolating part of the later inside the suberized tissue [23]. Thus, both parameters are dependent on phellogen activity, and the negative correlation found between them can be explained by the temporary cessation of phellogen activity, even in small localized regions, having a drastic impact on cork growth and depreciating its quality. Interestingly, we found one DNA methylation polymorphism (C4_487) that strongly correlates with low percentage of ‘nails’, *i*.*e*. high cork quality, and more stable phellogens. This result seems to indicate a potential role of cytosine methylation in the regulation of phellogen-localized death, which is also corroborated by the higher expression of *QsDMAP1* in low quality corks [39]. *QsDMAP1 is* a putative Methyltransferase 1 Associated Protein 1, homologous of the yeast chromatin remodelling complex SWR1 [65], shown to be involved in DNA methylation, DNA repair [66] and cell cycle control [67].

### DNA methylation may be involved in lenticular phellogen activity

A significant positive correlation was found between cork thickness and the area and length of cork pores. Previous studies about the effect of growth rate on cork structure already describe this correlation [64]. Pores are naturally found in all cork planks, however their number, dimension and distribution vary widely among trees [23,24,27], with high quality corks showing fewer and small diameter pores and bad quality corks showing the opposite pattern. Pores have been regarded as gas trade structures that exchange gases across the trunk [68] resembling stomata in terms of function. Indeed, the lenticular channels in the young virgin cork are formed below stomata [24] suggesting a common initial development regulation. Moreover, a recent study shows that bad quality cork-producing trees have higher expression of putative stomatal/lenticular-associated genes than good quality corks [31]. Regardless of lenticels development below stomata in the cork oak first periderm, in *amadia* corks stomata do not occur, although lenticular channels are present. Actually, phellogen originates two different types of cells to the outside: cells that either differentiate as suberized cork cells or, in a very small proportion, cells forming a loosely filling tissue, whose disaggregation leads to the lenticular channels or pores that radially cross the cork [24]. Similarly to the phellogen, the vascular cambium contains two types of morphologically distinct cell types—fusiform initials and ray initials that give rise to the axial and radial components of xylem, respectively [60]. The identity of cambial cells is rather determined by positional cues (reviewed in [69]) but the determination of phellogen cells’ identity and the molecular mechanisms underlying those differences are intriguing issues still to be uncovered. Thus, it is not known how or when phellogen cells fate is established. However, DNA methylation is likely involved in such differentiation as we found a MSAP sequence that is unmethylated in almost all corks with high pore area. Another particular fragment appears in two states: unmethylated in almost all corks with high pore area, and methylated in three quarters of the corks with small pores. These MSAP sequences and their methylation status seem to be good candidates to be used as markers for the pore area, which is one of the most important cork quality traits.

## Conclusions

This is the first work involving a genome-wide approach to correlate cork quality traits and DNA methylation in living cork cells. This study offers evidence that DNA methylation is associated with differences in cork cellular characteristics directly related to original or traumatic phellogen activity. The presence of cork quality trait-associated markers and their distribution across the three populations, support the hypothesis of a DNA methylation role in the regulation of cork quality traits. After confirmation of epigenetic status inheritance, further demonstration of a causative linkage between these methylation polymorphisms and cork quality traits will require additional investigation, namely by: (1) comparing with a reference genome to identify the sequence and genomic context of these markers, and their relationship with potential targets and gene expression, allowing to determine the methylation mechanisms underlying the phenotypic variation observed; (2) unravelling if these DNA methylation variants are independent of genetic variation by searching candidate genetic *loci* associated with them; (3) bisulphite-converted restriction site associated DNA sequencing (BsRADseq) [70] or whole epigenome analysis for a thorough genome DNA methylation study of the phellogen types producing contrasting cork qualities. This would allow mapping major DNA methylation differences, and finding other candidate *epiloci* associated with cork quality.

As final conclusion, our findings provide new tools to assess cork quality, and may still motivate further studies about the involvement of DNA methylation in altered forest trees phenotypes.

## Supporting Information

S1 Fig Example of a high quality cork showing small and few lenticular channels or pores. (PNG)

S2 Fig Example of a bad quality cork showing lignified phloem inclusions within the suberized tissue. (PNG)

S3 Fig Epigenetic differentiation in leaves and cork tissues from *amadia* cork-producing trees. (PNG)

S4 Fig Heatmap representing pairwise Pearson correlation coefficients between cork quality parameters measured for *amadia* cork planks from the three populations under study. Several parameters were measured for transversal (Tr), radial (R) and tangential (Ta) sections independently.
(TIF)

S1 Table List of oligonucleotides employed in MSAP analysis. (DOCX)

S2 Table Code for the primer combinations used in the selective amplification. (DOCX)

S3 Table MSAP markers raw data matrix for the three populations and both tissues. (XLSX)

S4 Table Cork quality traits assessed in *amadia* cork planks. (DOCX)

S5 Table Pairwise Pearson correlations between cork quality traits. (XLSX)
