# Supplementary figures and images for: Differential DNA Methylation Patterns Are Related to Phellogen Origin and Quality of Quercus suber Cork
Source: PLoS One. 2017 Jan 3;12(1):e0169018. doi: 10.1371/journal.pone.0169018 (PMC5207400; doi:10.1371/journal.pone.0169018)

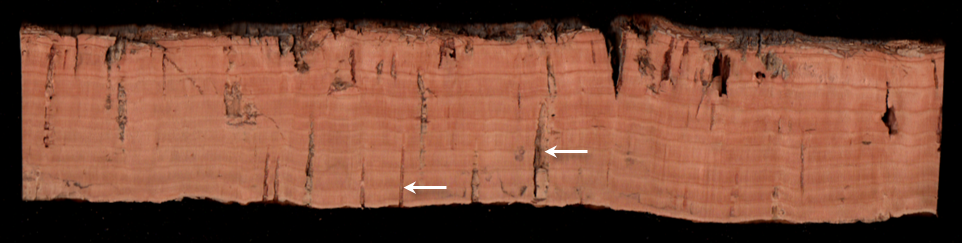

Supplement: S1 Fig — (PNG) [file pone.0169018.s001.png]

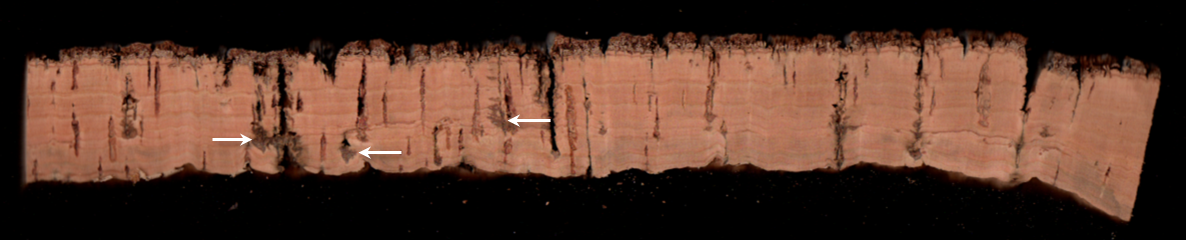

Supplement: S2 Fig — (PNG) [file pone.0169018.s002.png]

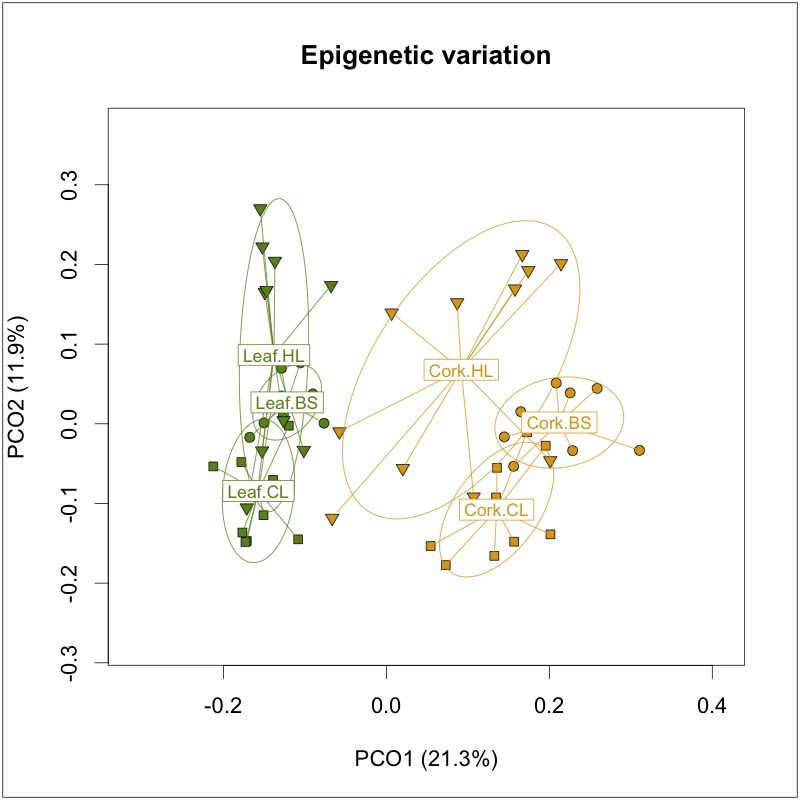

Supplement: S3 Fig — (PNG) [file pone.0169018.s003.png]

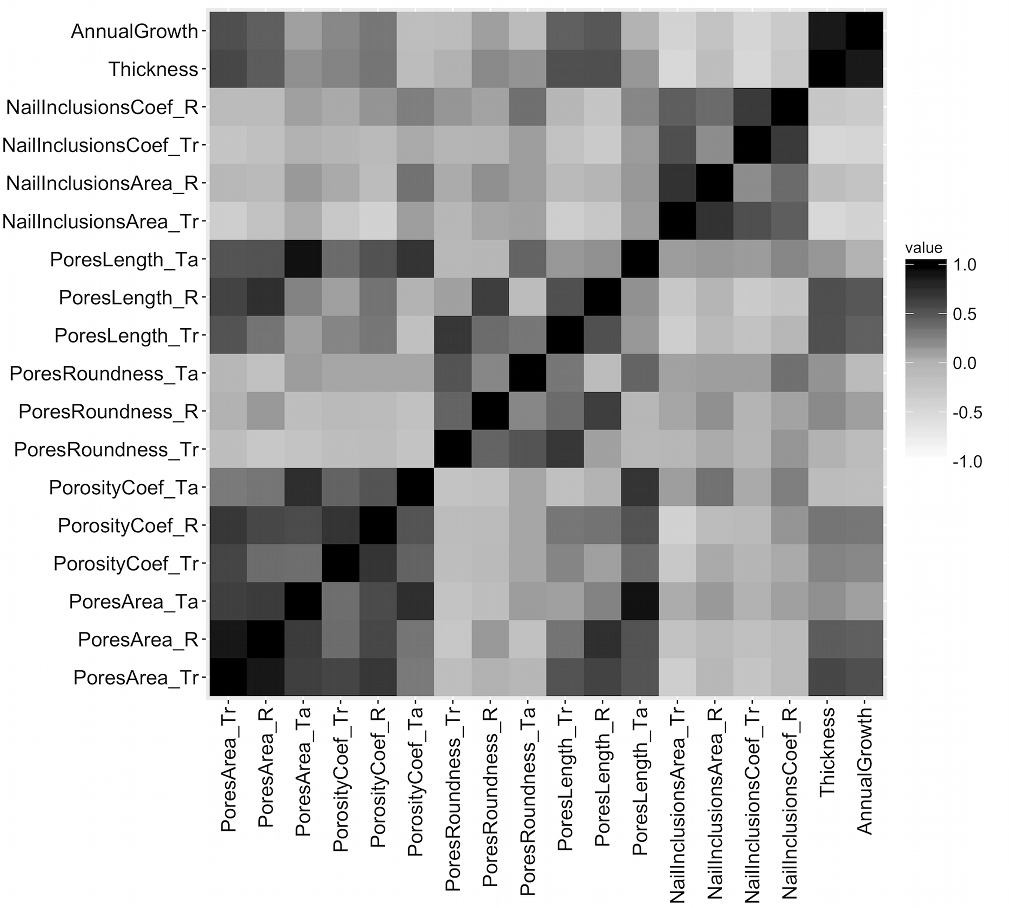

Supplement: S4 Fig — Several parameters were measured for transversal (Tr), radial (R) and tangential (Ta) sections independently. (TIF) [file pone.0169018.s004.tif]
