# Supplementary material for: Differential DNA Methylation Patterns Are Related to Phellogen Origin and Quality of Quercus suber Cork
Source: PLoS One. 2017 Jan 3;12(1):e0169018. doi: 10.1371/journal.pone.0169018 (PMC5207400; doi:10.1371/journal.pone.0169018)
Supplement: S1 Table — (DOCX) [file pone.0169018.s005.docx]

**S1 Table - Oligonucleotides employed in MSAP analysis**

| **Primer** | **Sequence** | **Enzyme** |
| --- | --- | --- |
| Eco_adaptor I | CTCGTAGACTGCGTACC | EcoRI |
| Eco_adaptor II | AATTGGTACGCAGTC | EcoRI |
| HM_adaptor I | GATCATGAGTCCTGCT | HpaII/MspI |
| HM_adaptor II | CGAGCAGGACTCATGA | HpaII/MspI |
| Pre_Eco (+A) | GACTGCGTACCAATTCA | EcoRI |
| Pre_HM | ATCATGAGTCCTGCTCGG | HpaII/MspI |
| Eco_ACA | GACTGCGTACCAATTCACA | EcoRI |
| Eco_ACG | GACTGCGTACCAATTCACG | EcoRI |
| Eco_AGG | GACTGCGTACCAATTCAGG | EcoRI |
| HM_TTA | ATCATGAGTCCTGCTCGGTTA | HpaII/MspI |
| HM_TCG | ATCATGAGTCCTGCTCGGTCG | HpaII/MspI |
