# Supplementary material for: Differential DNA Methylation Patterns Are Related to Phellogen Origin and Quality of Quercus suber Cork
Source: PLoS One. 2017 Jan 3;12(1):e0169018. doi: 10.1371/journal.pone.0169018 (PMC5207400; doi:10.1371/journal.pone.0169018)
Supplement: S2 Table — (DOCX) [file pone.0169018.s006.docx]

**S2 Table - Combinations of primers used in the selective amplification**

| **Code** | **Primers** |
| --- | --- |
| C1 | Eco-ACA-FAM + HM-TCG |
| C2 | Eco-ACG-FAM + HM-TCG |
| C3 | Eco-ACA-FAM + HM-TTA |
| C4 | Eco-AGG-FAM + HM-TTA |
