# Supplementary material for: Differential DNA Methylation Patterns Are Related to Phellogen Origin and Quality of Quercus suber Cork
Source: PLoS One. 2017 Jan 3;12(1):e0169018. doi: 10.1371/journal.pone.0169018 (PMC5207400; doi:10.1371/journal.pone.0169018)
Supplement: S4 Table — (DOCX) [file pone.0169018.s008.docx]

**S4 Table** - Average values (± SD) of cork quality traits assessed in transversal, radial and tangential sections of amadia cork planks

|  | **Barradas da Serra - BS** | | | **Companhia das Lezírias - CL** | | | **Herdade dos Leitões - HL** | | |
| --- | --- | --- | --- | --- | --- | --- | --- | --- | --- |
|  | **Transversal** | **Radial** | **Tangential** | **Transversal** | **Radial** | **Tangential** | **Transversal** | **Radial** | **Tangential** |
| Pores Area (mm^2^) ^a^ | 4.79 ± 1.22  (25.4%) | 5.91 ± 2.21  37.5%) | 3.68 ± 1.68  (45.6%) | 4.88 ± 1.49  (30.5%) | 5.70 ± 1.77  (31.1%) | 2.78 ± 0.97  (34.9%) | 4.97 ± 2.73  (54.9%) | 7.21 ± 4.40  (61.1%) | 2.90 ± 2.12  (73%) |
| % Porosity^a^ | 8.60 ± 1.44  (16.7%) | 7.64 ± 2.39  31.3%) | 7.61 ± 3.70  (48.7%) | 7.70 ± 3.18  (41.4%) | 8.01 ± 2.70  (33.7%) | 6.34 ± 1.65  (26%) | 7.43 ± 3.20  (43%) | 7.46 ± 2.96  (39.7%) | 5.80 ± 3.57  (61.6%) |
| Pores Roundness^b^ | 5.36 ± 1.25  (23.3%) | 4.31 ± 0.81  18.7%) | 2.66 ± 0.38  (14.1%) | 5.16 ± 0.89  (17.3%) | 3.88 ± 0.46  (11.9%) | 2.02 ± 0.33  (16.6%) | 3.64 ± 0.61  (16.9%) | 3.92 ± 0.73  (18.7%) | 1.77 ± 0.23  (12.9%) |
| Pores Length (mm)^ab^ | 5.87 ± 1.11  (19%) | 5.18 ± 0.96  (18.5%) | 3.07 ± 0.56  (18.1%) | 5.96 ± 1.13  (18.9%) | 5.27 ± 0.97  (18.4%) | 2.55 ± 0.32  (12.6%) | 4.95 ± 0.87  (17.6%) | 5.40 ± 1.09  (20.1%) | 2.51 ± 0.64  (25.6%) |
| Nail Area (mm^2^)^a^ | 3.45 ± 3.40 (98.6%) | 11.97 ± 21.13 (176.5%) | ND | 2.38 ± 1.89 (79.4%) | 4.91 ± 2.60 (53%) | ND | 2.83 ± 1.49 (52.8%) | 3.93 ± 2.92 (74.2%) | ND |
| % Nail^a^ | 0.65 ± 0.96  (150.1%) | 1.28 ± 1.70  (133.3%) | ND | 0.24 ± 0.39  (163%) | 0.78 ± 1.00  (129.9%) | ND | 0.63 ± 0.91  (144.5%) | 0.48 ± 0.35  (72.9%) | ND |
| Thickness (mm)^b^ | 32.71 ± 9.67  (29.6%) | | | 30.77 ± 8.61  (28%) | | | 31.32 ± 8.99  (28.7%) | | |
| Annual Growth (mm/year)^b^ | 3.02 ± 0.89  (29.7%) | |  | 3.72 ± 1.31  (35.3%) | |  | 3.68 ± 1.09  (29.5%) | |  |

^a^ Differences in means were compared using Kruskal-Wallis non-parametric and Dunn´s Multiple Correction post-hoc test (significance level = 0.05)

^b^ Differences in means were compared using one way ANOVA (significance level = 0.05)

Coefficient of variation (ratio of standard deviation to the mean) is indicated between brackets
